# Effects of educational practices on the peritonitis risk in peritoneal dialysis: a retrospective cohort study with data from the French peritoneal Dialysis registry (RDPLF)

**DOI:** 10.1186/s12882-020-01867-w

**Published:** 2020-05-29

**Authors:** Hélène Bonnal, Clémence Bechade, Annabel Boyer, Thierry Lobbedez, Sonia Guillouët, Christian Verger, Maxence Ficheux, Antoine Lanot

**Affiliations:** 1 Normandie Univ, UNICAEN, CUMR, CHU de Caen Normandie, Néphrologie, Avenue de la cote de nacre, 14033 Caen-Cedex, France; 2 U1086 INSERM – ANTICIPE – Centre Régional de Lutte Contre le Cancer François Baclesse, Caen, France; 3 RDPLF, 30 Rue Sere Depoin, 95 300 Pontoise, France

**Keywords:** Educational practices, Health literacy, Patient education, Peritoneal dialysis, Peritonitis

## Abstract

**Background:**

Peritoneal dialysis (PD) is a home-based therapy performed by patients or their relatives in numerous cases, and the role of patients’ educational practices in the risk of peritonitis is not well assessed. Our aim was to evaluate the effect of PD learning methods on the risk of peritonitis.

**Methods:**

This was a retrospective multicentric study based on data from a French registry. All incident adults assisted by family or autonomous for PD exchanges in France between 2012 and 2015 were included. The event of interest was the occurrence of peritonitis. Cox and hurdle regression models were used for statistical analysis to asses for the survival free of peritonitis, and the risk of first and subsequent peritonitis.

**Results:**

1035 patients were included. 967 (93%) received education from a specialized nurse. Written support was used for the PD learning in 907 (87%) patients, audio support in 221 (21%) patients, and an evaluation grid was used to assess the comprehension in 625 (60%) patients. In the “zero” part of the hurdle model, the use of a written support and starting PD learning with hands-on training alone were associated with a lower survival free of peritonitis (respectively HR 1.59, 95%CI 1.01–2.5 and HR 1.94, 95%CI 1.08–3.49), whereas in the “count” part, the use of an audio support and starting of PD learning with hands-on training in combination with theory were associated with a lower risk of presenting further episodes of peritonitis after a first episode (respectively HR 0.55, 95%CI 0.31–0.98 and HR 0.57, 95%CI 0.33–0.96).

**Conclusions:**

The various PD education modalities were associated with differences in the risk of peritonitis. Prospective randomized trials are necessary to confirm causal effect. Caregivers should assess the patient’s preferred learning style and their literacy level and adjust the PD learning method to each individual.

## ABREVIATIONS

PDPeritoneal dialysis

CCIC harlson comorbidity index

RDPLF French Peritoneal Dialysis Registry

BMI Body mass index

CAPD Continuous ambulatory peritoneal dialysis

APD Automated peritoneal dialysis

## Background

It is now well demonstrated that peritoneal dialysis (PD) delivers a high-quality treatment to patients presenting with end-stage renal disease. The survival of patients treated by PD is equivalent to the survival of those treated by hemodialysis, and several authors have shown that the technique is well tolerated and cost effective [1–3]. However, PD remains underused in several countries [4]. Peritonitis is a major cause of technique failure, and is one of the predominant factors limiting the extensive use of PD [5, 6]. The rate of peritonitis is highly variable worldwide, and within countries [7–9]. The risk of peritonitis depends on non-modifiable factors (age, sex, diabetes) and modifiable factors (anti-infectious prophylaxis, catheter care, assistance for PD) [10, 11]. It is important to accurately analyze the effect of modifiable factors, since they are the most relevant to decreasing the rate of peritonitis [12]. To our knowledge, there is a lack of data regarding the role of patients’ educational practices in the peritonitis risk [13]. Recommendations for PD training were proposed by the ISPD in 2016, but the adherence to these guidelines is probably far from optimal in France and worldwide [3, 14]. The objective of our study was to describe the educational practices proposed to French patients treated by PD and to evaluate the effect of these currently applied educational practices on the risk of peritonitis.

## Methods

This was an observational retrospective study using data from the French Language Peritoneal Dialysis Registry (RDPLF). The registry includes several optional moduli, one of which is the “nurses’ practices” modulus. Out of the 167 centers registered in the RDPLF, 94 participated to the “nurses’ practices” modulus. All patients registered in the “nurses’ practices” modulus of the RDPLF, starting PD between January 1, 2012 and December 31, 2015 were included in the study. The end of the observation period was December 31, 2016. Exclusion criteria were: assistance by nurse for PD exchanges, because the aim of the study was to assess the effect of educational practices dedicated to patients or their relatives, and age younger than 18 years when starting PD.

### Definition of variables

#### Individual characteristics

Age at PD initiation, the number of new patients per year per center, sex, body mass index (BMI), diabetes mellitus, the causal nephropathy, the presence of illiteracy learning disability, manual disability, hearing or visual impairment were extracted from the registry. Comorbidities were assessed by calculating a modified Charlson score, which is the Charlson score after subtracting the age sub score, to evaluate the role of the comorbidities independently of the patient’s age.

#### Characteristics of centers and treatments

PD modality (continuous ambulatory peritoneal dialysis (CAPD) or automated peritoneal dialysis (APD)), PD assistance (self-PD or family assisted PD), a previous treatment before PD initiation (hemodialysis, renal transplantation or no replacement therapy), and the administrative type of center (non-profit, general, university or private hospital) in which the patient was treated were listed.

#### Educational practices

Data about the educational practices used during the learning of PD of the patients were retrieved: the timing of the education regarding the catheter placement, whether the education had been provided by a nurse specialized in PD, the use of audio or written support for PD learning which was respectively an audio file, or a printed booklet given to the patient to learn about, the assessment of the patients’ knowledge and skills concerning PD with an evaluation grid, defined as a standard list of items used for assessment of the patients’ knowledge and skills concerning PD prior or after the learning courses (of note, standardized within each center but not between all centers), the use of standard theoretical courses or adapted theoretical courses (adapted to the presumed preferred learning style of patients), and the start of learning with theory or with hands-on training.

#### Events of interest

The number of peritonitis for each patient was retrieved, with the declared cause, and the responsible germs.

### Statistical analysis

Categorical variables were described by proportion and percentage, and continuous variables were described by their median value and first and third quartile. Overlapping of different educational practices were presented using an upset diagram [15].

All statistical models were performed at the individual levels. The event of interest was the first peritoneal infection after the start of PD. A survival analysis with a Cox proportional hazard model was assessed, to estimate the association between the covariates and the risk of first peritonitis. The proportional hazard assumption was tested graphically by inspection of the Schoenfeld residual plots. The Cox model allows the assessment of the survival free of peritonitis, but does not consider the risks of multiples peritonitis cases. A classic Poisson regression model is commonly used to analyze count data, but it assumes that the number of patients with zero event is not overrepresented and that the variance of the distribution of the number of peritonitis cases is equal to the mean. Whenever these assumptions are violated, alternative models should be considered. Therefore, we used a hurdle model, which is built with two parts: a “zero count” rate ratio modeling the risk of presenting one peritonitis during the follow-up period, and a “count” rate ratio modeling the number of peritonitis during the follow-up period for the patients who have had a first episode. The exposure time to PD was used as an offset in both parts of the hurdle model.

Covariates associated with the risk of first peritonitis with *p* < 0.2 in a bivariate analysis were entered in the multivariate analysis, and educational practices were entered a priori since they were the covariates of interest. The existence of collinearity between covariates was assessed by the generalized variance inflation factor.

In a sensitivity analysis, we assessed survival models focusing on the peritonitis due to handled germs (cocci Gram positive germs excluding *Enterococcus* and *Streptococcus agalactiae*), because this type of peritonitis is mainly due to contamination while handling PD fluid exchange, and therefore, good education should be associated with a lower risk.

To describe the distribution of the different practices in the centers, we arbitrarily chose a cut-off of 75% to decide whether a practice was standardized or not in the PD center. In other words, whenever a practice was used in more than 75% of the registered cases of the center, the practice was estimated standardized in the center. This value of 75% was chosen because it was estimated sufficient to make sure that whenever a practice was used 75% or more of the time in a given center, it would not be due to chance, but it would be a standard practice of the center. In the center-level analysis (and only in this analysis), 22 centers were excluded because fewer than 5 patients were registered. The center-level analysis was strictly descriptive, and no statistical tests were performed at the center-level.

Fewer than 10% of the data were missing in the dataset. Therefore, we performed a complete case analysis.

Statistical analyses were performed with R 3.5.1 (R foundation for statistical computing) including the survival and lme4 packages.

The RDPLF has the approval of the French National Ethics Committee (*Commission nationale de l’informatique et des libertés*) with the agreement number 542668 and fulfills the GDPR requests. This study took place within the framework of this authorization. In the extracted dataset, information was anonymized by the RDPLF.

This study was reported in accordance with the Strengthening the Reporting of Observational Studies in Epidemiology (STROBE) guidelines [16].

## Results

**Fig. 1:**
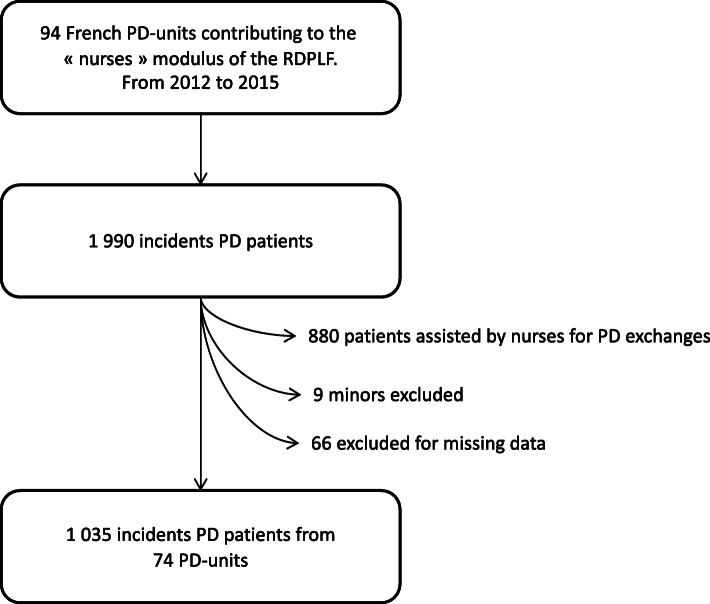
Flowchart of the study

During the study period, there were 1990 incident PD patients registered in the “nurses’ practices” modulus of the RDPLF, in 94 PD-units. A total of 880 patients were assisted by a nurse for PD exchange and were therefore excluded. Nine patients were excluded because they were under 18 years of age, and 66 were excluded because of missing data. Finally, data concerning 1035 patients from 74 PD units were analyzed (Fig. 1). The median follow-up time was 15.2 months. At the center level, the median number of incident PD patients per year was 5.6 (interquartile range (IQR) 3.4–9.4).

### Univariate analysis

#### Study population

The median age was 59 (IQR 45–69), and 667 (64%) patients were male. Of the 1305 patients included, 937 (91%) were autonomous and a family caregiver assisted 98 (9%) patients. The three main causes of nephropathy were glomerulonephritis in 227 (22%) patients, vascular nephropathy in 198 (19%) patients, and diabetic nephropathy in 129 (12%) patients. Two hundred and forty-two (23%) patients were diabetic. The median BMI was 25 kg/m^2^ (IQR 22–28). Seven hundred and seventy (74%) patients were treated with neither dialysis nor renal transplantation before the PD initiation. One hundred and seventy-eight (17%) patients presented deafness and/or visual impairment, and 145 (14%) patients had learning disability. The characteristics of the population are displayed in (Table 1).

**Table 1:** Population’s characteristics

	All patients (***N*** = 1035)	
**Covariates**	**Median (IQR)**	
**Age at PD initiation (years)**	59 (45–69)	
**BMI (kg/m**^**2**^**)**	25 (22–28)	
	**N**	**%**
**Sex (Male)**	667	64
**Diabetes**	242	23
**Nephropathy**		
Polycystic kidney disease	124	12
Glomerulonephritis	227	22
Systemic disease	44	4
Diabetic nephropathy	129	12
Interstitial nephritis	60	6
Vascular	198	19
Uropathy	32	3
Other cause	100	10
Unknown	121	12
**Previous treatment**		
No RRT	770	74
Hemodialysis	197	19
Transplantation	19	7
**PD modality (APD)**	628	61
**Modality of PD assistance**		
Self PD	937	91
Family assisted PD	98	9
**Type of center**		
General hospital	487	47
Non profit	260	25
University hospital	181	17
Private	107	10
**Charlson’s score**		
< 3	487	47
3	173	17
4	130	13
> 4	245	24
**Functional impairment**		
No impairment	857	83
Hearing impairment	41	4
Visual impairment	126	12
Hearing and visual impairment	11	1
**Manual disability**	46	4
**Illiteracy**	14	1
**Learning disability**	145	14

*IQR* Inter-quartile range, *PD* Peritoneal dialysis, *BMI* Body mass index, *APD* Automated peritoneal dialysis

#### Educational practices

PD education was performed before catheter placement in 988 (95%) patients, and PD education was provided by a specialized nurse in 967 (93%) patients. The use of written support was widely spread in 907 (88%) patients. Theory and hands-on training were proposed simultaneously to start PD education in 666 (64%) patients. The distribution of educational practices at the patient’s level is synthesized in (Table 2).

**Table 2:** Distribution of educational practices at the patient’s level and at the center’s level

Covariates		All patients (***N*** = 1035)		Number of centers (***N*** = 53)	
				Practice used in more than 75% of cases in the center	
		N	%	N	%
**Delay between education and catheter placement**	More than 30 days prior	335	32.37	2	3.77
	Within 30 days	653	63.09	17	32.07
	After catheter placement	47	4.54	0	0
**Education provided by specialized nurse**	Yes	967	93.43	48	90.57
	No	68	6.57	1	1.89
**Use of a written support**	Yes	907	87.63	42	79.25
	No	128	12.37	3	5.66
**Use of an evaluation grid**	Yes	625	60.39	26	49.06
	No	410	39.61	16	30.19
**Use of an audio support**	Yes	221	21.35	2	3.77
	No	814	78.65	40	75.47
**Theory learning**	No	14	1.35	0	0
	Standardized	47	4.54	0	0
	Adapted	974	94.11	47	88.67
**Education started with**	Theory	269	25.99	5	9.43
	hands-on training	100	9.66	1	1.88
	Both hands-on training and theory	666	64.35	25	47.17

A practice was arbitrarily defined as standardized in a given center if the practice was used in more than 75% of the registered cases of the center

**Fig. 2:**
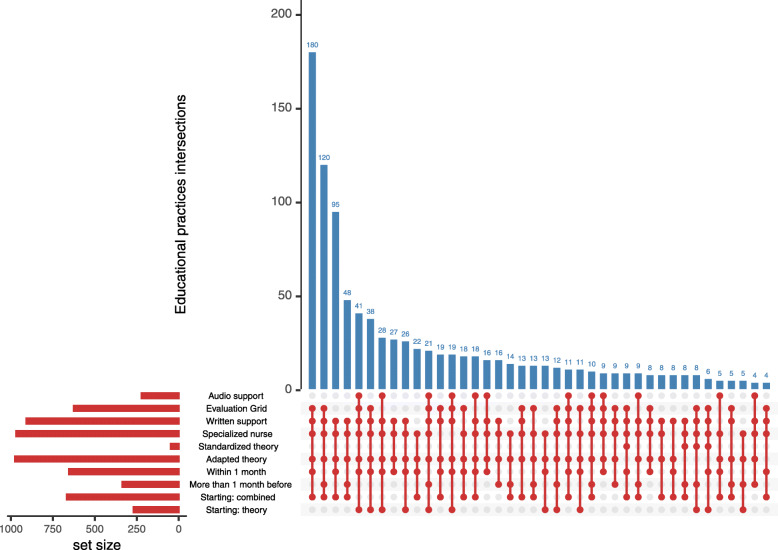
Upset diagram representing the overlapping between educational practices. “Within 1 month” and “More than 1 month before” correspond to the timing of PD learning compared with catheter placement. “Starting: combined” and “Starting: theory” are respectively the starting of PD learning with theory and hands-on training at the same time, and theory alone

Table 2 also shows the distribution of educational practices standardly used at the center level (Table 2). A nurse specialized in PD provided the PD education to patients in every center. A written support was standardly used in 42 (79%) centers. Only two (4%) centers used an audio support during the PD education of their patients. The more frequent association pattern of educational practices was the use of an evaluation grid, use of a written support, learning provided by a PD-specialized nurse, starting with adapted theory and hands-on training, and learning started within a month prior to catheter placement. The frequencies of these associations are depicted on the (Fig. 2).

#### Characteristics of the peritonitis

There were 565 episodes of peritonitis occurring in 339 patients. The peritonitis rate during the study period was 0.34 episodes per patient per year at risk. One hundred and sixty-three (48%) peritonitis were due to Gram-positive cocci, and 80 (24%) were due to Gram-negative bacilli. Causes were registered for the first peritonitis only. The major registered cause was asepsis mistake in 108 (31.86%) cases of peritonitis, endogenous contamination in 47 (13.86%) cases, tunnel infection in 11 (3.24%) cases, mechanical issue in 11 (3.24%) cases, and unknown in 145 (42.77%) cases. Table 3 synthesizes the characteristics of peritonitis (Table 3). The proportions of peritonitis according to the educational practices are presented in Table 4, according to the number of peritonitis: 0, one or more than two (Table 4).

**Table 3:** Characteristics of the peritonitis

	All patients (*N* = 1315)	
	N	%
**Number of peritonitis**		
0	702	67.83
1	207	20
2	71	6.86
3	31	3
4	12	1.16
5 or more	12	1.16
**Type of germ for first peritonitis**		
Gram negative bacilli	80	23.60
Gram positive bacilli	17	5.01
Gram negative Cocci	1	0.29
Gram positive Cocci	163	48.08
Mycobacteria and unknown	78	23.01
**Causes for first peritonitis**		
Asepsis mistake	108	31.86
Endogenous contamination	47	13.86
Mechanical issue	11	3.24
Tunnel infection	11	3.24
Cat	6	1.77
Nosocomial	3	0.88
Icodextrine	2	0.59
Unknown	151	44.54

**Table 4:** Proportion of peritonitis according to the different educational practice

Number of peritonitis	0 (***N*** = 943)	1 (***N*** = 236)	2 or more (***N*** = 136)
**Timing of education regarding catheter placement**			
More than 30 days prior to catheter placement	225 (32%)	65 (31%)	45 (36%)
Within 30 days prior to catheter placement	444 (63%)	132 (64%)	77 (61%)
After catheter placement	33 (5%)	10 (5%)	4 (3%)
**Education provider**			
Non-specialized nurse	48 (7%)	13 (6%)	7 (6%)
Specialized nurse	654 (93%)	194 (94%)	119 (94%)
**Use of written support**	609 (87%)	182 (88%)	116 (92%)
**Use of an evaluation grid**	424 (60%)	125 (60%)	76 (60%)
**Use of audio support**	156 (22%)	40 (19%)	25 (20%)
**Theory learning**			
No	10 (1%)	3 (1%)	1 (1%)
Adapted learning	660 (94%)	191 (92%)	123 (98%)
Standardized learning	32 (5%)	13 (6%)	2 (2%)
**First step of education**			
Theory	194 (28%)	38 (18%)	37 (29%)
Hands-on training	63 (9%)	26 (13%)	11 (9%)
Theory and hands-on training	445 (63%)	143 (69%)	78 (62%)

*HR* Hazard ratio, *95% CI* 95% Confidence interval, *PD* Peritoneal dialysis, *BMI* Body mass index, *CAPD* Continuous ambulatory peritoneal dialysis, *APD* Automated peritoneal dialysis

### Cox model

#### Bivariate analysis

**Fig. 3:**
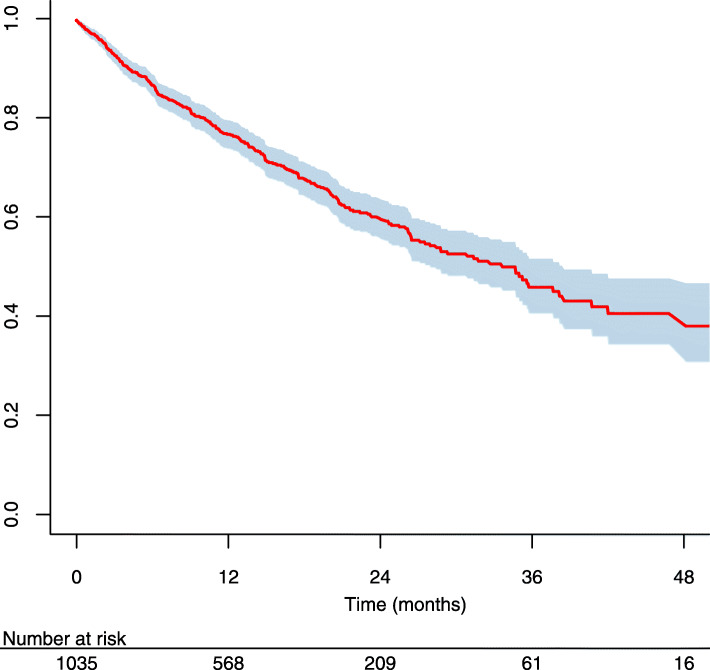
Kaplan-Meier curve for peritonitis-free survival and 95% confidence interval

The results of the bivariate Cox model are shown in additional material (Additional file 1). The patient specific covariates that were significantly associated with the risk of peritonitis were: having a BMI between 25 and 30 kg/m^2^ (HR 1.37, 95%CI 1.08–1.74), and between 30 and 35 kg/m^2^ (HR 1.56, 95%CI 1.10–2.22) compared to the class of reference 18 to 25 kg/m^2^, having been treated with hemodialysis prior to PD compared to patients naive of prior extra renal epuration treatment (HR 1.48, 95%CI 1.15–1.91), a modified Charlson’s score equal to 3 compared with a score of 2 as the reference (HR 1.38, 95%CI 1.04–1.83), and starting PD learning with hands-on training alone or in combination with theory (respectively HR 1.51, 95%CI 1.02–2.23 and HR 1.29, 95%CI 1.00–1.68). No significant association was retrieved between the risk of peritonitis and the other educational practices tested. The Kapplan Meier curve for peritonitis free survival is shown in (Fig. 3).

#### Multivariate analysis

**Fig. 4:**
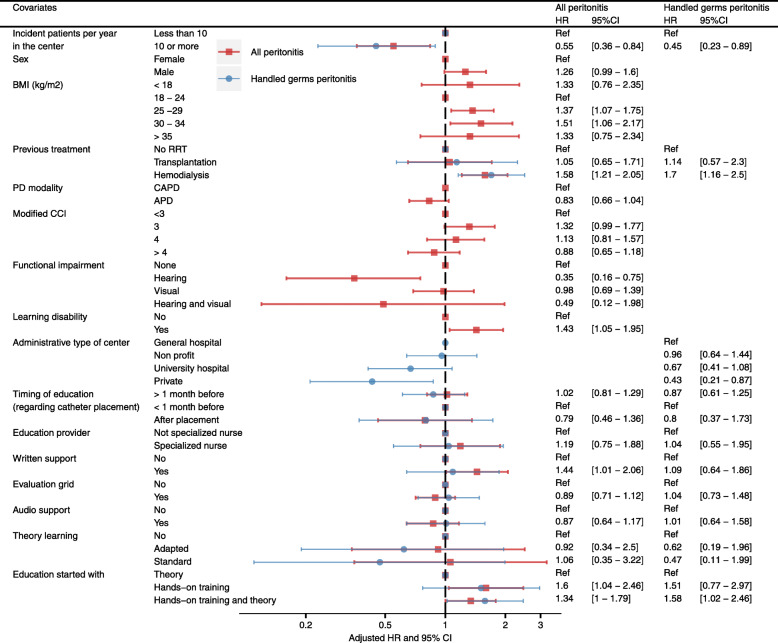
Multivariate Cox model for peritonitis-free survival due to all germs, and to handled germs (individual-level analysis). HR: hazard ratio, 95%CI: 95% confidence interval, BMI: body mass index, RRT: renal replacement therapy, PD: peritoneal dialysis, CCI: Charlson comorbidity index, CAPD: continuous ambulatory PD, APD: automated PD. Handled germs are cocci Gram positive germs excluding *Enterococcus* and *Streptococcus agalactiae*.

The results of the Cox multivariate model are shown in (Fig. 4). Baselines covariates associated with a protective effect against peritonitis were being treated in a PD unit with ten or more incident patients per year (HR 0.55, 95%CI 0.36–0.84), presence of a hearing impairment (HR 0.35, 95%CI 0.16–0.75). Some other covariates were associated with a higher risk of peritonitis: BMI comprised between 25 and 30 kg/m^2^ (HR1.37, 95%CI 1.07–1.75) or 30 and 35 kg/m^2^ (HR 1.51, 95%CI 1.06–2.17), having been treated with hemodialysis prior to PD (HR 1.58, 95%CI 1.21–2.05), and presenting a learning disability (HR 1.43, 95%CI 1.05–1.95). Two educational variables were significantly associated with a higher risk of peritonitis: use of a written support (HR 1.44, 95%CI 1.01–2.06) and starting education with hands-on training alone or combined with theory (respectively HR 1.6 95%CI 1.04–2.46 and HR 1.34, 95%CI 1.02–2.46).

### Hurdle model

#### Bivariate analysis

In the “count” part of the hurdle model, starting PD between 30 and 49 years of age or between 50 and 64 years of age were associated with a higher risk of having further peritonitis after a first episode (respectively HR 5.13, 95%CI 1.36–19.33, and HR 4.39, 95%CI 1.18–16.40). On the other hand, being treated in a center with ten or more new patients per year (HR 0.36, 95%CI 0.14–0.97), having a BMI greater than 35 kg/m2 (HR 0.04, 95%CI 0–0.37), and starting PD learning with theory and hands-on training concomitantly were protective (HR 0.36, 95%CI 0.14–0.92).

In the “zero” part of the hurdle model, male sex (HR 1.26, 95%CI 1.00–1.58), a BMI between 25 and 30 or between 30 and 35 kg/m2 (respectively HR 1.34, 95%CI 1.05–1.71, and HR 1.59, 95%CI 1.11–2.27), previous treatment by hemodialysis (HR 1.55, 95%CI 1.19–2.01), a modified Charlson comorbidity index equal to 3 (HR 1.41, 95%CI 1.05–1.89), and starting PD education with hands-on training alone or in combination with theory (respectively HR 1.64, 95%CI 1.01–1.72 and HR 1.32, 95%CI 1.10–2.45) were significantly associated with the risk of presenting a first episode of peritonitis, whereas hearing impairment was protective (HR 0.44, 95%CI 0.21–0.93) (Additional file 2).

#### Multivariate analysis

**Fig. 5:**
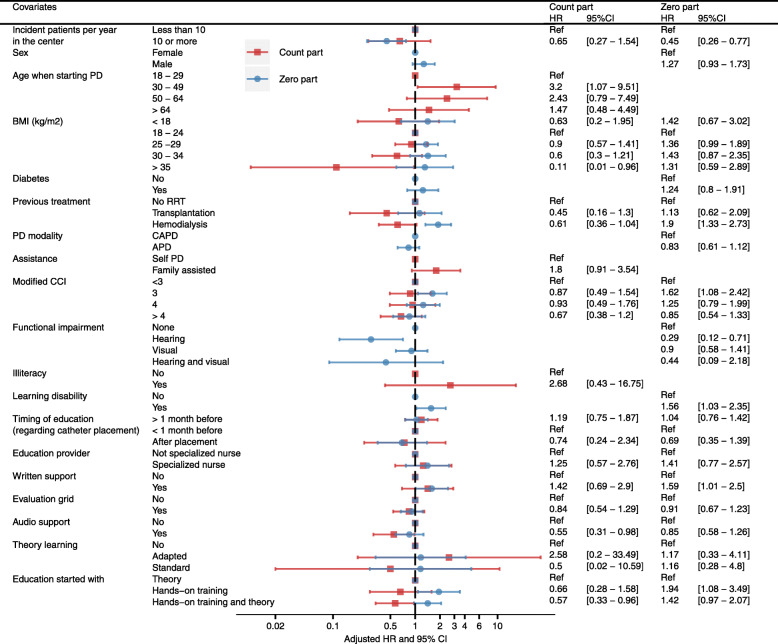
Multivariate hurdle model for risk of peritonitis (individual-level analysis). The “count” part assesses for the risk of presenting further peritonitis after a first episode, and the “zero” part assesses for the risk of presenting a first episode of peritonitis. HR: hazard ratio, 95%CI: 95% confidence interval, BMI: body mass index, RRT: renal replacement therapy, PD: peritoneal dialysis, CCI: Charlson comorbidity index, CAPD: continuous ambulatory PD, APD: automated PD.

After adjustment in the “count” part of the hurdle model, a BMI greater than 35 kg/m2 (HR 0.11, 95%CI 0.01–0.96), a previous treatment by hemodialysis (HR 0.11, 95%CI 0.01–0.96), use of an audio support for PD learning (HR 0.55, 95%CI 0.31–0.98) and starting PD education concomitantly with both hands-on training and theory (HR 0.57, 95%CI 0.33–0.96) were protective factors, whereas starting PD between 30 and 49 years of age (HR 3.2, 95%CI 1.07–9.51) was associated with a higher risk of presenting further peritonitis after a first episode (Fig. 5).

In the “zero” part of the multivariate hurdle model, previous treatment by hemodialysis (HR 1.9, 95%CI 1.33–2.73), a modified Charlson comorbidity index equal to 3 (HR 1.62, 95%CI 1.08–2.42), learning disability (HR 1.56, 95%CI 1.03–2.35), use of a written support for PD learning (HR 1.59, 95%CI 1.01–2.5), and starting PD education with hands-on training (HR 1.94, 95%CI 1.08–3.49) were significantly associated with the risk of presenting a first episode of peritonitis, whereas being treated in a center with ten or more new patient per year (HR 0.45, 95%CI 0.26–0.77), and the presence of hearing impairment were protective (HR 0.29, 95%CI 0.12–0.71) (Fig. 5).

### Sensitivity analysis

One hundred and fifty-three peritonitis were due to handled germs. The results of the Cox multivariate analysis are shown in (Fig. 4). Starting PD education with hands-on training and theory at the same time was associated with a significantly higher risk of peritonitis due to manipulated germs (HR 1.58, 95%CI 1.02–2.46).

## Discussion

In this large observational study of patients autonomous or assisted by close relatives for PD care, we found that the use of a written support during PD learning and starting PD learning with hands-on training alone were associated with a lower survival free of peritonitis, whereas the use of an audio support and starting of PD learning with hands-on training in combination with theory were associated with a lower risk of presenting further episodes of peritonitis after a first episode.

PD is a home-based therapy; therefore, it is widely known that the patients and caregivers’ education is essential to ensure a smooth running of the treatment. The best way to achieve this education is not well defined currently. However, several authors have tried to identify training methods to prevent peritonitis, and some protective factors have been pointed out. In Uruguay, Gadola et al. have shown that a PD education program based on the adult-learning principles was associated with a lower peritonitis rate (0.29 per patient-year vs 0.48 per patient-year with a previous PD education program, *p* < 0.05) [17]. In a study from the Brazilian registry it was demonstrated that a cumulative training time greater than to 15 h was associated with significantly different peritonitis rates (0.26 per year at risk vs 0.32 per year at risk, *p* = 0.01) compared with a cumulative training time of less than 15 h. Less than 1 h of training/day was associated with a higher incidence rate of peritonitis when compared with the intervals of 1–2 h/day (*p* = 0.03). Training in the immediate 10 days after implantation of the catheter was associated with the highest peritonitis rate compared with training prior to catheter implantation (0.32 per year vs 0.28 per year) [18]. The location of PD training could have an impact on peritonitis rates, according to the results from a monocentric Spanish study where the peritonitis incidence decreased from 1 episode /24.5 patient/month to 1 episode /44.4 patient/month after having implemented the training sessions in the patient’s home [19]. Hsu et al. demonstrated that a multidisciplinary predialysis education was associated with a longer peritonitis free survival (HR 0.59, 95%CI 0.43–0.81) [20].

Learning PD theory and hands-on training can be challenging for some patients, and the PD learning programs should therefore be adapted to each patient. Several authors have shown that educational factors were associated with PD complications. In a large multicentric Brazilian observational study, Martin et al. showed that the patients’ educational level was associated with the risk for survival free of peritonitis (illiteracy versus higher education level: HR 1.75, 95%CI 1.04–2.92, elementary versus higher education level: HR 1.64, 95%CI 1.06–2.54 and secondary versus higher education level: HR 1.57, 95%CI: 0.99–2.49) [21]. An association between lower education level and the risk for peritonitis (HR 1.45 95% CI 1.01–2.06, *p* = 0.0454) was observed in a Taiwanese monocentric study in 2013 [22]. Kim et al. demonstrated similar results in a Korean population, where an education level of middle school or lower (academic year < 9) was associated with a significant risk for peritonitis (HR 1.61, 95%CI 1.10–2.36) [23]. Special attention could therefore be paid to the patients with lower education levels, who may need adapted learning styles. Assessing the knowledge and behavior in 191 Italian PD patients, Russo et al. found out that 47% of patients needed retraining, especially those in the early or late phase of PD therapy [24].

Congruently, we have found with the Cox model and the zero part of the hurdle model that hearing impairment was associated with a lower risk of presenting a first episode of peritonitis (respectively HR 0.35, 95%CI 0.16–0.75 and HR 0.29, 95%CI 0.12–0.71). One might wonder how a disability could be protective. We make the hypothesis that in subjects with hearing impairments, the PD learning could be better individualized, with better adaptation to the abilities and learning patterns of the subjects. This protective effect however was not found in visual impaired patients, but it appears reasonable to think that a visual impairment is more disabling than a hearing one for realization of the PD exchange, due to the required dexterity.

The ISPD proposed recommendations for teaching PD to patients in 2016 [14]. These guidelines mainly advised basing the training programs on the principles of adult education, and proposed a five-day program of approximatively 3 h per day. Suggestions were made to assess the patient’s preferred learning style and to implement teaching style accordingly. The use of the VARK (Visual, Auditory, Read and write, and Kinesthetic) learning style questionnaire was proposed.

To our knowledge, the effect of the teaching medium on PD learning has not been studied. We tried to assess the effect of using several educational supports on the risk of peritonitis. We found the use of a written support to be associated with a lower survival free of peritonitis. It has been shown that the information materials aimed at patients with chronic kidney disease are above the average patient’s literacy level, therefore many patients do not take advantage of the written supports provided, because they lack the skills required to understand them [25]. Moreover, we hypothesize that in some cases, written support may be provided to the patients with fewer explanations and/or fewer investments from the caring team in the learning process, therefore leading to poorer quality of the care. However, this result should be taken cautiously because it is not robust to the sensitivity analysis on handled germs peritonitis.

Patients who received audio support for PD learning experienced fewer recurrences after a first peritonitis episode. Notably, the use of audio support is not widespread in dialysis centers with only 2 centers using it as a standard feature, whereas written supports were used as standard features in 42 centers, as shown in Table 2. Therefore, we suppose that patients who benefit from audio learning may have been selected on some individual characteristics. This individualization of the educational support could be the explanation for this better outcome.

These results appear to be in line with the ISPD recommendations to assess the patient’s preferred learning style and to plan the education accordingly. No support may be universally better for PD learning. PD training programs should contain different supports to be able to deliver the best education for each given patient.

Starting PD learning with hands-on training was associated with shorter survival free of peritonitis. The combination of hands-on training and theory were associated with a lower risk of further peritonitis after a first episode whereas it was nearly significantly associated with a higher risk for first episode in the “zero” part of the hurdle model. This phenomenon has been called a “dissonant effect” by Lachenbruch [26]. A telling example is considering an antibiotic treatment, that could be effective in reducing the risk of carrying some specific bacteria, whereas it could be associated with the growth of these bacteria after a first infection, due to antibiotic resistance. A possible explanation for the tendencies observed here could be that patients who started PD learning with hands-on training get more confident earlier with a risk of being less attentive to the rules of asepsis. After a first complication, they should become more cautious and present fewer recurrence.

It is worth noticing that counterintuitively, in the “count” part of the multivariate hurdle model, BMI greater than 35 kg/m2 was associated with a lower risk of presenting further peritonitis after a first episode. Obesity has been considered as a relative contra-indication to PD [27]. Therefore, we can hypothesize that nephrologists would be more likely to propose transfer to hemodialysis after a first peritonitis episode in obese patients, whereas they would have pursued the technique for non-obese patients. The protective effect against repeated peritonitis found here would in fact be biased by the presence of transfer to hemodialysis which is a competing event regarding peritonitis. This shorter time to transfer to hemodialysis therapy for obese patients has been reported in a 2017 American study [28].

Another worth commenting result was the higher risk for peritonitis of previous treatment by hemodialysis in the “zero” part of the multivariate hurdle model, whereas it was near significantly a protective effect in the “count” part. This dissonant effect can be explained by the fact that patients who experimented hemodialysis prior to PD might be transferred more easily than the others for several reasons: presence of a functional vascular access for hemodialysis, wish from the patient and/or the nephrologist to get back to a known dialysis modality. The lower survival risk free of peritonitis in patients previously treated with hemodialysis has already been found in a former study with data from the French registry, using a multivariate Cox model (cs-HR 1.15, 95%CI 1.03–1.29) [29]. In a Polish study, Liberek et al. have shown that patients treated with hemodialysis prior to PD had a significantly higher peritonitis rate compared with those in whom PD was the initial method of renal replacement therapy (0.86 vs 0.62 episode per year, *p* < 0.05) [30]. Patients are transferred from hemodialysis to PD because of complications such as vascular access problems, hemorrhage risk, or cardiovascular condition making hemodialysis no more pursuable. In such patients, PD is not the modality of choice so their involvement may be poorer, explaining the higher peritonitis risk.

Our study is genuine due to the covariates we studied. We assessed the effect of modifiable factors, which is always relevant because the results of the study may have clinical implications. We used quality data extracted from a nationwide registry known for its quality. These strengths should be balanced with some limitations. Some bias may exist due to the retrospective type of the study. The population is not representative of the whole French PD population because some PD centers did not participate in the “nurses’ practices” modulus in which the educational covariates were collected. Furthermore, we did not know how the methods of education for PD learning were chosen, which could have led to some selection bias. It would be of interest to assess and adjust the analysis on the educational and socioeconomic status of the patients, because these factors might interact with the educational method chosen and their effect.

## Conclusion

We have found that the modality of PD learning was associated with the risk of peritonitis in autonomous or family assisted PD patients. The use of a written support and starting PD learning with hands-on training alone were associated with a lower survival free of peritonitis, whereas the use of an audio support and starting of PD learning with hands-on training in combination with theory were associated with a lower risk of presenting further episodes of peritonitis after a first episode. According to the ISPD guidelines, caregivers should assess the patient’s preferred learning style and their literacy, to best adjust the PD learning program according to these preferences and skills, and offering individualized learning methods. Further interventional studies focusing on educational practices are needed to accurately determine the best practice in PD learning for patients.

## Supplementary information

**Additional file 1 Table 1** Cox model for survival free of peritonitis. Bivariate analysis. Table showing the results of the bivariate Cox survival model for survival free of peritonitis.

**Additional file 2 Table 2.** Hurdle model for survival free of peritonitis. Bivariate analysis. Table showing the results of the bivariate hurdle model for the risk of peritonitis

## Data Availability

The datasets used and analyzed during the current study are available from the corresponding author on reasonable request after approval obtained from the RDPLF.
