# Supplementary material for: Effects of educational practices on the peritonitis risk in peritoneal dialysis: a retrospective cohort study with data from the French peritoneal Dialysis registry (RDPLF)
Source: BMC Nephrol. 2020 May 29;21:205. doi: 10.1186/s12882-020-01867-w (PMC7260816; doi:10.1186/s12882-020-01867-w)
Supplement: Supplementary file 1 — Additional file 1 Table 1 Cox model for survival free of peritonitis. Bivariate analysis. Table showing the results of the bivariate Cox survival model for survival free of peritonitis. [file 12882_2020_1867_MOESM1_ESM.docx]

Additional Table 1: Cox model for survival free of peritonitis. Bivariate analysis.

| **Covariates** | **HR** | **95% CI** |
| --- | --- | --- |
| **Number of new patients per year in the center** |  |  |
| Fewer than 10  Ten or more | Ref.  0.70 | -  0.47-1.05 |
| **Age at PD initiation (years)** | | |
| 18-29  30 – 49  50 – 64  > 65 | Ref  0.99  1.02  0.92 | -  0.63-1.57  0.65-1.59  0.59-1.44 |
| **Sex (Male)** | 1.25 | 0.99-1.56 |
| **BMI (kg/m^2^)** | | |
| <18  18 - 25  25 - 30  30 - 35  > 35 | 1.27  Ref  1.37  1.56  1.46 | 0.74-2.21  -  1.08-1.74  1.10-2.22  0.84-2.52 |
| **Diabetes** | 1.17 | 0.92-1.50 |
| **Nephropathy** | | |
| Polycystic kidney disease  Glomerulonephritis  Systemic disease  Diabetic  Interstitial nephritis  Vascular  Other cause  Unknown  Uropathy | Ref.  0.68  0.72  1.02  0.83  0.79  0.73  0.81  1.16 | -  0.46-1.00  0.37-1.39  0.68-1.53  0.49-1.39  0.54-1.15  0.46-1.16  0.53-1.24  0.61-2.20 |
| **Treatment before PD initiation** | | |
| No dialysis  Transplantation  Hemodialysis | Ref  1  1.48 | -  0.62-1.59  1.15-1.91 |
| **PD modality** |  |  |
| CAPD  APD | Ref  0.84 | -  0.68-1.05 |
| **Modality of PD assistance** | | |
| Self PD  Family assisted PD | Ref  0.80 | -  0.54-1.20 |
| **Type of center** | | |
| General hospital  Non profit  University hospital  Private | Ref  0.92  0.84  0.73 | -  0.71-1.20  0.62-1.14  0.50-1.08 |
| **Modified Charlson’s score** | | |
| 2  3  4  >5 | Ref  1.38  1.27  0.99 | -  1.04-1.83  0.92-1.75  0.74-1.31 |
| **Manual disability** | 1.02 | 0.61-1.71 |
| **Functional impairment** |  |  |
| No impairment  Hearing impairment  Visual impairment  Hearing and visual impairment | Ref.  0.42  0.95  0.45 | -  0.20-0.89  0.68-1.33  0.11-1.80 |
| **Illiteracy** | 0.85 | 0.27-2.65 |
| **Learning disability** | 1.27 | 0.95-1.71 |
| **Timing of education regarding catheter placement** |  |  |
| More than 30 days prior to catheter placement  Within 30 days prior to catheter placement  After catheter placement | 1.01  Ref.  0.86 | 0.80-1.26  -  0.50-1.47 |
| **Education provider** |  |  |
| Non-specialized nurse  Specialized nurse | Ref.  1.06 | -  0.68-1.65 |
| **Use of written support** | 1.29 | 0.91-1.82 |
| **Use of an evaluation grid** | 0.91 | 0.73-1.13 |
| **Use of audio support** | 0.83 | 0.64-1.09 |
| **Theory learning** |  |  |
| No  Adapted learning  Standardized learning | Ref.  1.01  1.08 | -  0.38-2.71  0.36-2.23 |
| **First step of education** |  |  |
| Theory  Hands-on training  Theory and hands-on training | Ref  1.51  1.29 | -  1.02-2.23  1.00-1.68 |

HR: hazard ratio; 95% CI: 95% Confidence interval; PD: Peritoneal dialysis; BMI : Body mass index ; CAPD : Continuous ambulatory peritoneal dialysis ; APD : automated peritoneal dialysis

Additional table 2: Hurdle model for survival free of peritonitis. Bivariate analysis

|  | **Zero part** | | **Count part** | |
| --- | --- | --- | --- | --- |
| **Covariates** | **HR** | **95% CI** | **HR** | **95% CI** |
| **Number of new patients per year in the center** |  |  |  |  |
| Fewer than 10  Ten or more | Ref.  0.73 | -  0.49-1.10 | Ref.  0.36 | -  0.14-0.97 |
| **Age** | | |  |  |
| 18-29  30 – 49  50 – 64  > 65 | Ref.  0.96  0.95  0.88 | -  0.60-1.53  0.60-1.50  0.56-1.38 | Ref.  5.13  4.39  1.74 | -  1.36-19.33  1.18-16.40  0.47-6.40 |
| **Sex (Male)** | 1.26 | 1.00-1.58 | 0.89 | 0.50-1.59 |
| **BMI (kg/m2)** | | |  |  |
| <18  18 - 25  25 - 30  30 - 35  > 35 | 1.34 Ref.  1.34  1.59  1.46 | 0.76-2.35  -  1.05-1.71  1.11-2.27  0.83-2.56 | 0.37  Ref.  0.79  0.51  0.04 | 0.09-1.44  -  0.43-1.44  0.22-1.20  0-0.37 |
| **Diabetes** | 1.19 | 0.93-1.53 | 0.79 | 0.43-1.47 |
| **Nephropathy** | | |  |  |
| Polycystic kidney disease  Glomerulonephritis  Systemic disease  Diabetic  Interstitial nephritis  Vascular  Other cause  Unknown  Uropathy | Ref.  0.68  0.70  1.04  0.84  0.82  0.76  0.74  1.18 | -  0.46-1.01  0.36-1.37  0.68-1.57  0.50-1.44  0.56-1.21  0.48-1.22  0.48-1.16  0.61-2.30 | Ref.  0.67  0.23  0.70  1.57  0.99  0.32  0.71  0.07 | -  0.25-1.74  0.03-1.61  0.24-2.02  0.41-6.00  0.37-2.64  0.09-1.12  0.24-2.10  0.01-0.42 |
| **Treatment before PD initiation** | | |  |  |
| No dialysis  Transplantation  Hemodialysis | Ref.  1.05  1.55 | -  0.65-1.68  1.19-2.01 | Ref.  0.46  0.69 | -  0.13-1.66  0.36-1.33 |
| **PD modality** |  |  |  |  |
| CAPD  APD | Ref.  0.85 | -  0.68-1.06 | Ref.  1.38 | -  0.80-2.40 |
| **Modality of PD assistance** | | |  |  |
| Self PD  Family assisted PD | Ref.  0.79 | -  0.53-1.19 | Ref.  2.03 | -  0.79-5.23 |
| **Type of center** | | |  |  |
| General hospital  Non profit  University hospital  Private | Ref.  0.98  0.92  0.76 | -  0.75-1.29  0.68-1.25  0.51-1.12 | Ref.  0.99  0.90  0.86 | -  0.52-1.89  0.41-2.00  0.33-2.23 |
| **Modified Charlson’s score** | | |  |  |
| 2  3  4  >5 | Ref.  1.41  1.29  1.03 | -  1.05-1.89  0.93-1.79  1.03-1.38 | Ref.  0.96  0.55  0.63 | -  0.45-2.04  0.26-1.16  0.31-1.28 |
| **Manual disability** | 1.07 | 0.64-1.82 | 0.57 | 0.13-2.42 |
| **Functional impairment** |  |  |  |  |
| No impairment  Hearing impairment  Visual impairment  Hearing and visual impairment | Ref.  0.44  0.91  0.49 | -  0.21-0.93  0.65-1.28  0.12-1.98 | Ref.  2.49  1.84  0 | -  0.21-28.83  0.78-4.34  0.00- >100 |
| **Illiteracy** | 0.79 | 0.25-2.48 | 6.19 | 0.54-70.99 |
| **Learning disability** | 1.25 | 0.93-1.69 | 0.90 | 0.43-1.87 |
| **Delay between education and catheter placement** |  |  |  |  |
| More than 30 days prior to catheter placement  Within 30 days prior to catheter placement  After catheter placement | 1.00  Ref.  0.81 | 0.79-1.26  -  0.47-1.41 | 1.15  Ref.  0.43 | 0.64-2.06  -  0.11-1.73 |
| **Education provider** |  |  |  |  |
| Non-specialized nurse  Specialized nurse | Ref.  1.14 | -  0.72-1.81 | Ref.  1.93 | -  0.72-5.18 |
| **Written support** | 1.32 | 0.93-1.88 | 2.03 | 0.86-4.77 |
| **Use of an evaluation grid** | 0.91 | 0.72-1.13 | 0.87 | 0.51-1.50 |
| **Use of audio support** | 0.83 | 0.63-1.10 | 1.03 | 0.52-2.04 |
| **Theory learning** |  |  |  |  |
| No  Adapted learning  Standardized learning | Ref.  1.13  1.08 | -  0.42-3.08  0.35-3.32 | Ref.  3.79  0.29 | -  0.21-69.40  0.01-8.88 |
| **First step of education** |  |  |  |  |
| Theory  Hands-on training  Theory and hands-on training | Ref.  1.64  1.32 | -  1.01-1.72  1.10-2.45 | Ref.  0.59  0.36 | -  0.31-1.11  0.14-0.92 |

HR: hazard ratio; 95% CI: 95% Confidence interval; PD: Peritoneal dialysis ; BMI : Body mass index ;s CAPD : Continuous ambulatory peritoneal dialysis ; APD : automated peritoneal dialysis.
